# Knockout mouse models as a resource for the study of rare diseases

**DOI:** 10.1007/s00335-023-09986-z

**Published:** 2023-05-09

**Authors:** Patricia da Silva-Buttkus, Nadine Spielmann, Tanja Klein-Rodewald, Christine Schütt, Antonio Aguilar-Pimentel, Oana V. Amarie, Lore Becker, Julia Calzada-Wack, Lillian Garrett, Raffaele Gerlini, Markus Kraiger, Stefanie Leuchtenberger, Manuela A. Östereicher, Birgit Rathkolb, Adrián Sanz-Moreno, Claudia Stöger, Sabine M. Hölter, Claudia Seisenberger, Susan Marschall, Helmut Fuchs, Valerie Gailus-Durner, Martin Hrabě de Angelis

**Affiliations:** 1 Institute of Experimental Genetics, German Mouse Clinic, Helmholtz Zentrum München, German Research Center for Environmental Health, Ingolstaedter Landstrasse 1, Neuherberg, Germany; 2 Institute of Experimental Genetics, Applied Computational Biology, Helmholtz Zentrum München, German Research Center for Environmental Health, Ingolstaedter Landstrasse 1, Neuherberg, Germany; 3 Institute of Developmental Genetics, Helmholtz Zentrum München, German Research Center for Environmental Health, Ingolstaedter Landstrasse 1, Neuherberg, Germany; 4 German Center for Diabetes Research (DZD), Ingolstaedter Landstrasse 1, 85764 Neuherberg, Germany; 5 Chair of Experimental Genetics, TUM School of Life Sciences, Technische Universität München, Alte Akademie 8, 85354 Freising, Germany; 6 Institute of Molecular Animal Breeding and Biotechnology, Gene Center, Ludwig-Maximilians-Universität München, Feodor-Lynen Strasse 25, 81377 Munich, Germany

## Abstract

Rare diseases (RDs) are a challenge for medicine due to their heterogeneous clinical manifestations and low prevalence. There is a lack of specific treatments and only a few hundred of the approximately 7,000 RDs have an approved regime. Rapid technological development in genome sequencing enables the mass identification of potential candidates that in their mutated form could trigger diseases but are often not confirmed to be causal. Knockout (KO) mouse models are essential to understand the causality of genes by allowing highly standardized research into the pathogenesis of diseases. The German Mouse Clinic (GMC) is one of the pioneers in mouse research and successfully uses (preclinical) data obtained from single-gene KO mutants for research into monogenic RDs. As part of the International Mouse Phenotyping Consortium (IMPC) and INFRAFRONTIER, the pan-European consortium for modeling human diseases, the GMC expands these preclinical data toward global collaborative approaches with researchers, clinicians, and patient groups.

Here, we highlight proprietary genes that when deleted mimic clinical phenotypes associated with known RD targets (*Nacc1, Bach2, Klotho alpha*). We focus on recognized RD genes with no pre-existing KO mouse models (*Kansl1l, Acsf3, Pcdhgb2, Rabgap1, Cox7a2*) which highlight novel phenotypes capable of optimizing clinical diagnosis. In addition, we present genes with intriguing phenotypic data (*Zdhhc5, Wsb2*) that are not presently associated with known human RDs.

This report provides comprehensive evidence for genes that when deleted cause differences in the KO mouse across multiple organs, providing a huge translational potential for further understanding monogenic RDs and their clinical spectrum. Genetic KO studies in mice are valuable to further explore the underlying physiological mechanisms and their overall therapeutic potential.

## Introduction

A rare disease (RD) is distinguished by its very low prevalence amid the population. Depending on the country, a disease is considered rare if it affects less than 5 in 10,000 people in the European Union (EU)(https://eur-lex.europa.eu/legal-content/EN/TXT/PDF/?uri=CELEX:32000R0141&from=EN),(Rodwell C. 2014) or fewer than 200,000 people in USA (USA, Orphan Drug Act of 1983). In a recent systematic global RDs review, Richter et al. (Richter et al. 2015) identified an average international prevalence of 1:2,500 people suffering from RDs. This accumulates to an estimated 27 to 36 million (6–8%) affected RD patients in the EU and 30 million in the USA, according to Rodwell (2014) (EC.Europa.EU). These are remarkable numbers when compared to a common disease like diabetes, where 34.1 million adult Americans aged 18 years or older (10% of the population) have been diagnosed (https://www.cdc.gov., National Diabetes Statistics Report, 2020–CDC).

Approximately 7,000 RDs have been described, of which 6,053 are annotated with prevalence or incidence information in Orphanet (reported January 2022), (https://rarediseases.info.nih.gov/diseases/pages/31/faqs-about-rare-diseases) (Haendel et al. 2020), however, only a few hundred have any treatment at present (https://www.orpha.net/orphacom/cahiers/docs/GB/Prevalence_of_rare_diseases_by_alphabetical_list.pdf). Diagnosis of RDs is challenging for clinicians due to extremely heterogeneous manifestations and low prevalence. Making the right diagnosis is therefore a long journey. Moreover, treatment, even after a diagnosis, is often unavailable and only about 5% of RDs have an approved medication by the US Food and Drug Administration (FDA). At present, 7,877 disease-gene relationships are annotated (http://www.orphadata.org). Most of them have genetic confounders (72%), some are cancers, infectious diseases or toxic, with the majority being congenital (70%) (Nguengang Wakap et al. 2020) (Rodwell 2014, EC.Europa.EU).

The German Mouse Clinic (GMC) was the first large-scale and comprehensive mouse phenotyping center worldwide. GMC nominates genes and presently produces single-gene knockout (KO) mutant mouse lines using transgenic technology (CRISPR). The KO mice go through a standardized, broad-based *in vivo* testing, using modern technologies with high translational impact, such as acoustic startle chambers, dual-energy X-ray absorptiometry (DXA), micro computed tomography (µCT), magnetic resonance imaging (MRI), auditory brainstem response (ABR), indirect calorimetry, optical coherence tomography (OCT), and echocardiography with ultra-high frequency ultrasound (Vevo3100). This enables the GMC to generate rich sets of important physiological data in areas of behavior, bone and cartilage development, neurology, clinical chemistry, haematology, immunology, allergy, energy metabolism, eye development and vision, cardiovascular morphology and function and pathology to further investigate candidate genes for human diseases. Detailed information on all the KO mouse lines and respective phenotypes are accessible at www.mouseclinic.de/results/phenomap-and-results/index.html. The GMC, as a partner of the International Mouse Phenotyping Consortium (IMPC), provides a relevant gene-based, data-rich resource for every gene-phenotype association to unravel the mammalian genome (Brown and Moore 2012). The KO mouse data are valuable to the research, genetic and medical communities involved in studying complex human diseases (Rosenthal and Brown 2007) as well as RDs (Investigators et al. 2021). In this context, (Seaby et al. 2021) have highlighted the contribution of mouse models in connecting target genes to large genomic datasets as part of a “gene-to-patient” approach. The establishment of such synergistic efforts with clinician and patient groups, improves the characterization of monogenic RDs, with the potential to accelerate novel areas for therapeutic exploitation (Brown 2021).

In this report, we present a selection of genes that, when deleted, show a broad phenotypic spectrum across different organ systems. Firstly, there are mouse KOs that are considered to mimic some of the clinical characteristics associated with known RD genes (*Nacc1, Bach2, Klotho alpha*). Secondly, there are genes with no pre-existing KO mouse models (*Kansl1l, Acsf3, Pcdhgb2, Rabgap1, Cox7a2*), which can be used to optimize clinical diagnosis. Lastly, we uncover genes (*Zdhhc5, Wsb2*) with previously unknown functions and disease associations that may represent future RD models.

It is timely to present to the scientific community the GMC mouse resource’s ability to gain further/novel insight into monogenic RDs by using KO mouse data. This noteworthy resource may be essential for improved RD comprehension, and offers an opportunity to discover yet unknown therapeutic approaches (e.g., dietary intervention or drug therapy) beneficial for patients. International collaborative research efforts are indispensable for improving RD diagnoses and treatments (Julkowska et al. 2017) (Monaco et al. 2022).

## Materials and methods

Briefly, mouse models were produced using the IMPC targeting strategy with CRISPR/Cas9 or ES cell technology (https://www.mousephenotype.org/understand/the-data/alleledesign/) at Helmholtz Munich (Fuchs et al. 2011) (Fuchs et al. 2018). ES cell technology enables visualization of the knocked out gene due to beta-galactosidase expression via the lacZ cassette. Each mutation was created and maintained on a standardized inbred C57BL/6N strain (for details see the page at the IMPC portal https://www.mousephenotype.org/data/search). The mice were genotyped to confirm the mutation in agreement with a genotyping protocol. Heterozygous mice were inter-crossed to generate homozygous and control mice for phenotypic analysis. For homozygous viable lines, the homozygote animals (7 m/7f) were comprehensively examined applying a standardized phenotyping pipeline. Mice were housed in individually ventilated cages (IVC) with water and standard mouse chow according to the directive 2010/63/EU, German laws and GMC housing conditions (https://www.mouseclinic.de/about-gmc/mouse-husbandry/index.html). All tests were approved by the district government of Upper Bavaria. Unless stated in the text, the mutant mice of the different mouse models and their corresponding C57BL/6N wild-types (WT) were between 8 and 16 weeks of age when broad-based phenotyped at the GMC as previously described (Fuchs et al. 2018) (www.mouseclinic.de). High-throughput phenotyping protocols for the GMC pipeline were in agreement with the IMPC standard procedures (https://www.mousephenotype.org/impress/PipelineInfo?id=14).

### Statistical analyses

Phenotypic data were analyzed and visualized using automated R-scripts (version 3.6.3) implemented in a database called “MausDB” (Maier et al. 2008). Depending on parameter distribution, tests for genotype effects were performed using Wilcoxon rank sum test, analysis of variance (ANOVA) with post-hoc Tukey honestly significant difference (HSD) test and / or linear models. Where necessary, body weight was included as confounder. For categorical data, a Fisher’s exact test was used. For each normally distributed parameter, mean and standard deviation, whereas for non-normally distributed data, median, 25th percentiles and 75th percentiles were calculated. A *p*-value < 0.05 was used as level of significance in the comparison of phenotypic observations between KO and WT mice; a correction for multiple testing was not performed. All abnormal phenotypic data were annotated based on the Mammalian Phenotype Ontology (MP) and on the Mouse Pathology Ontology (MPATH) tools. All data are publically and freely available on the GMC (www.mouseclinic.de/results/phenomap-and-results/index.html) and on the IMPC portal (https://www.mousephenotype.org/data/search?term=&type=gene). Please note that export of data from *Klotho *and *Cox7a2* homozygotes, but not heterozygotes, to IMPC are not possible due to the mismatch of standard operating procedures (SOPs). After each gene results section, a *“New findings from the GMC”* section was introduced to summarize results generated from the phenotyping procedures at the GMC, which were not previously described in the scientific literature.

## Results

Within the GMC, single-gene KO mice were generated and systematically analyzed covering a broad range of tests to identify novel phenotypes. All KO mutants described were homozygous for a particular gene (Table 1). Wild-type (WT) mice of the same background strain (C57BL/6N) served as healthy controls. Up to now, there are 517 lines characterized in the GMC and a couple of representative examples classified by the RD gene association are highlighted in this report. Detailed information on all the lines and phenotypes can be found here: www.mouseclinic.de/results/phenomap-and-results/index.html. Here, it is important to note that we are presenting GMC data, where parameters from mutant homozygotes were compared with in parallel-analyzed controls and statistically evaluated. These data are therefore not identical to those on the publicly accessible IMPC site caused by differences in study analysis (e.g., accumulation of controls).

**Table 1:** Description of known RD genes associated with human RD name and ORPHA code, genes with no pre-existing KO mouse models and genes with previously unknown functions and RD associations. Special emphasis on new phenotypes generated by the GMC, which are described for the first time in this report

Grouping	Gene	Rare disease*	ORPHA	Previously published KO mouse model	Mouse model of homolog gene or family	GMC KO mouse model phenotypes: new findings
Known RD genes	*Nacc1*	Neurodevelopmental disorder with epilepsy, cataracts, feeding difficulties, and delayed brain myelination (het)	ORPHA:500,545	Yes	No	Viable mice** after weaning, renal
	*Bach2*	Chronic myeloid leukemia	ORPHA:521	Yes	No	Failure to thrive, eye
	*Klotho alpha*	Familial hyperphosphatemic tumoral calcinosis	ORPHA:306,661	Yes	No	Histological evidence of diverse tissue calcifications
Known RD genes with no pre-existing KO mouse models	*Kansl1l*	Koolen-de vries syndrome	ORPHA:96,169	No	Yes but Kansl1 -not viable	Viable mice**, in-depth eye
	*Acsf3*	Combined malonic and methylmalonic aciduria	ORPHA:289,504	No	No	Renal, Immuno
	*Pcdhgb2*	Prune belly syndrome	ORPHA:2970	No	Yes but whole Pcdh-gamma cluster (but mice did not survive) or exon combinations	Viable mice**, anemia, cardio
	*Rabgap1*	Warburg micro syndrome 1	ORPHA:2510	No	No but Tbc1d20 KO	Histological evidence of decreased corpus callosum
	*Cox7a2*	Fatal infantile cardioencephalomyopathy due to cytochrome C oxidase deficiency	ORPHA:1561	No	Yes but Cox7a-rl (Cox7a2l) and Cox2a1 KO	Growth failure, histological evidence of altered brown adipocytes
Previously unreported RD gene candidates: Novel mouse models (potential new gene targets)	*Zdhhc5*	No associated RD	–	No	ZDHHC5-GT (gene trap)	Dysmorphology, eye
	*Wsb2*	No associated RD	–	No	No	Dysmorphology, eye, renal, heart, male infertility

*Disorder association as per https://www.genecards.org/

**Mice were viable postnatal past 21 days

### Known rare disease genes: KO mouse models

#### *Nacc1* (Nucleus accumbens associated 1)

**Fig. 1:**
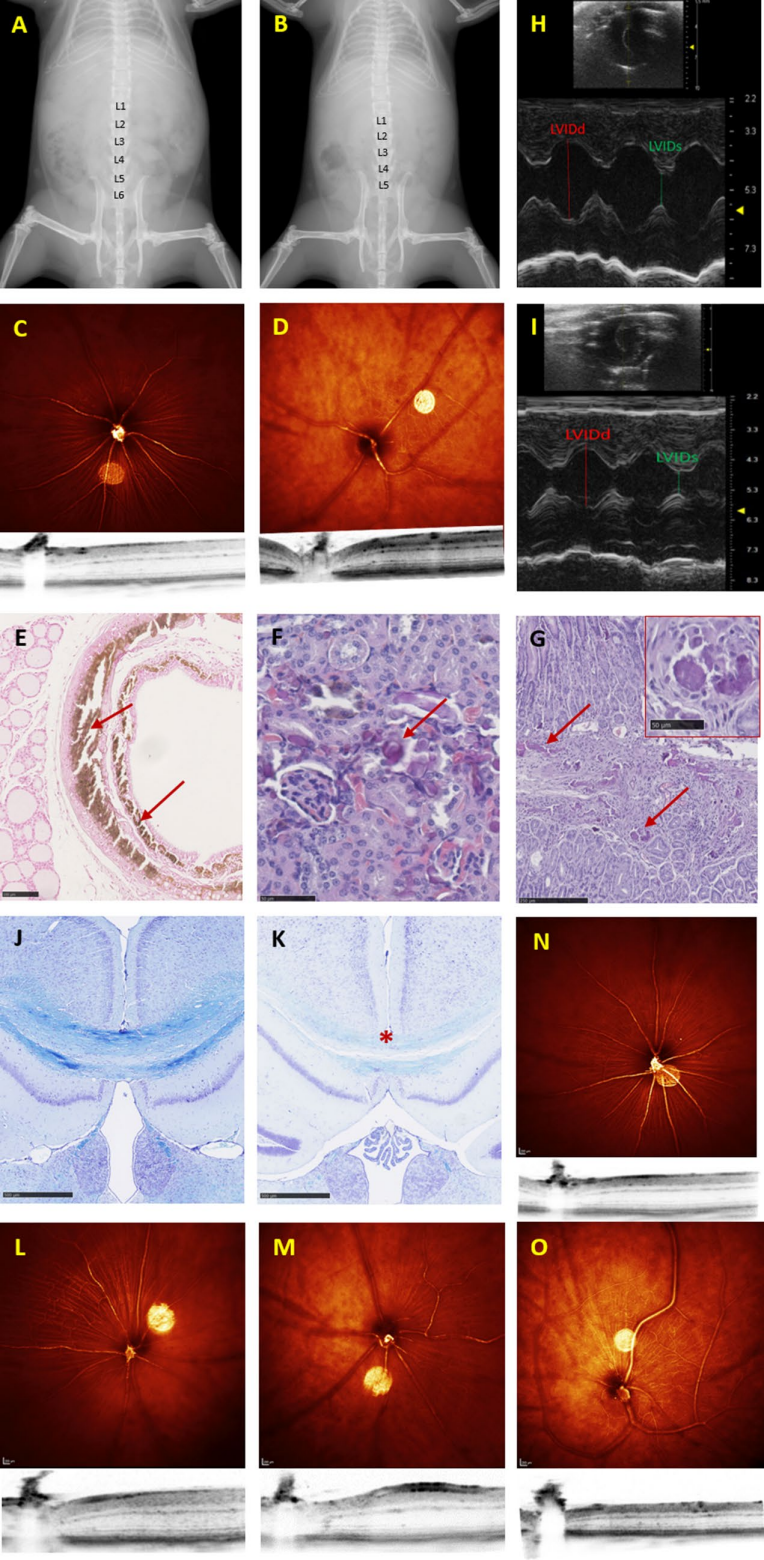
Representative high-throughput phenotyping findings from the GMC for selected KO mouse models. Panel A and B: X-ray image analysis shows a decrease in the number of lumbar vertebrae in *Nacc1* KO (**B**) in comparison with WT (**A**) mice. The phenotype was present in 3/3 female and 5/5 male mutants analyzed. Panel C and D: Optical coherence tomography with optic disc (OD) cupping, fewer blood vessel and OD alteration in *Bach2* KO (**D**) mice and normal OD in WT (**C**). Panel E, F and G: Microphotographs of Von Kossa-stained trachea (**E**) and haematoxylin and eosin-stained kidney (**F**) and glandular stomach (**G**) sections illustrate the marked tissue calcification in *Klotho alpha* KO mice. Panel H and I: Transthoracic echocardiograms (short-axis, m-mode) depict decreased left ventricular internal diameter (LVID) in diastole (d, red) and systole (s, green) in *Kansl1l* KO (**I**) compared to WT mice (**H**). Panel J and K: Microphotographs of Luxol Fast Blue-stained coronal brain sections show the reduced thickness of the corpus callosum (*) in *Rabgap1* KO mice (**K**) compared to WT (**J**). Panel L and M: Optical coherence tomography show fewer blood vessel development and reduced retinal thickness in *Rabgap1* KO mice (**M**) compared to WT (**L**). Panel N and O: Optical coherence tomography show reduced number of fundic main blood vessels and reduced total retinal thickness in *Wsb2* KO mice (**O**) compared to WT mice (**N**). All WT were of C57BL/6N background

In-depth characterization of the *Nacc1* KO mouse model revealed a significant decrease in body weight (*p* < 0.001, MP:0001262) in KO compared to WT mice. Interestingly, there was an absence of vibrissae in the majority of female mutant mice. X-ray imaging unveiled a decrease in number of lumbar vertebrae (MP:0004645) in all mutant mice analyzed (Fig. 1 Panel A and B). In addition, there was a decreased bone mineral content (*p* < 0.001, MP:0010124) and density (*p* < 0.001, MP:0,000,063) in KO compared to WT mice. Mutant mice showed decreased locomotor activity and grip strength (*p* < 0.001) and increased threshold for auditory brainstem response indicative of impaired hearing ability (MP:0001419, MP:0010053, and MP:0011967, respectively). Their behavior was also different from WT with decreased rearing activity in open field (*p* = 0.002, MP:0001420) and decreased acoustic startle response/sensorimotor recruitment (*p* < 0.001, MP:0001489). In mutant mice, there was a decreased total retina thickness (*p* < 0.001, MP:0011965). Glucose tolerance tests indicated impaired glucose tolerance (*p* < 0.001, MP:0005293) in mutants in both sexes. Blood clinical chemistry showed decreased plasma potassium (*p* = 0.009, MP:0005628), creatinine (*p* = 0.014, MP:0005554), phosphate (*p* = 0.001, MP:0000198), and total iron binding capacity (*p* = 0.021) levels in mutant male mice compared to control mice, whereas female KO and WT mice showed similar levels. Interestingly, there were other genotype-related differences with clear sexual dimorphism, namely, increased plasma cholesterol (*p* = 0.027, MP:0005178), glucose (*p* = 0.046, MP:0001559), urea (*p* = 0.05, MP:0005565), alkaline phosphatase level (p = 0.025, MP:0002968), and lipase (*p* = 0.034, MP:0011886) levels in male mutant mice. Macrocytosis (*p* = 0.001, MP:0000248) was observed in both sexes. By transthoracic echocardiography (TTE), reduced stroke volume (*p* = 0.015, MP:0011952) and cardiac output (*p* = 0.004, MP:0003393) were observed in mutant mice. Furthermore, the aortic diameter was decreased (*p* = 0.007, MP:0010997) in the male mutant mice. Spleen weight was reduced (*p* = 0.05, MP:0004953) in mutant mice and plasma IgE levels decreased in both sexes (*p* = 0.001, MP:0002492). Histopathological examination revealed that some mutant mice had renal morphological abnormalities (MP:0002135) (bilateral hydronephrosis (MP:0000519), infarct, protein casts) accompanied by a significant increase in plasma urea. In addition, one female mutant mouse developed a fluid-filled uterus (hydrometra, MP:0009709).

*New findings from the GMC*: The *Nacc1* KO mouse model from the GMC was viable until 16 weeks of age and showed a renal phenotype manifested by renal plasma markers and histology.

#### *Bach2* (BTB and CNC homology 1, basic leucine zipper transcription factor 2)

The *Bach2* KO mice were clearly underweight (*p* < 0.001, MP:0001262) compared with WT mice. There were, associated with this, signs of reduced mineral bone content (*p* < 0.001, MP:0010124) and density (*p* < 0.001, MP:0010119) as well as tibia length (*p* = 0.05, MP:0002764) in mutant mice. Additionally, the KO mice showed hypoactivity (*p* = 0.009, MP:0031392) and limb grasping (*p* < 0.001, MP:0001513) in the modified SmithKline Beecham, Harwell, Imperial College, Royal London Hospital, phenotype assessment (SHIRPA) protocol. Irrespective of sex, mutant mice showed increased distance traveled (*p* = 0.009, MP:0001399) with decreased rearing activity (*p* < 0.001, MP:0002757) and extended center time and distance in the open field assay. Prepulse inhibition and acoustic startle reactivity were reduced (*p* = 0.006, MP:0009142 and *p* < 0.001, MP:0001488, respectively) in comparison with control mice. The *Bach2* mutant mice had decreased plasma glucose (p = 0.001, MP:0000189), cholesterol (*p* = 0.003, MP:0005179), triglycerides (*p* = 0.001, MP:0,002,644), and creatinine (*p* = 0.004, MP:0005554) levels and increased unsaturated transferrin levels, a marker of iron binding capacity (*p* = 0.004, MP:0011896). Also they showed increased liver enzyme activities (alkaline phosphatase (ALP), *p* = 0.002, MP:0002968, alanine aminotransferase (ALT), *p* < 0.001, MP:0002941, aspartate aminotransferase (ASAT), *p* < 0.001, MP:0005343) compared to controls. Flow cytometry analysis of the spleen revealed a significant increase in production of most myeloid cell lineages (*p* < 0.001, MP:0013663), T cells (*p* < 0.001, MP:0005015), and natural killer (NK) (*p* < 0.001, MP:0008039) cells. In contrast, B cell frequency was reduced (*p* < 0.001, MP:0005017). Additionally, splenocytes from *Bach2* KO mice, showed impairing in the proliferation rate under in vitro induction of immunoglobulin class switching (CSR) when compared with control mice. *Bach2* KO mice were found to have affected optic nerve disc morphology (*p* = 0.041, MP:0008259, Fig. 1 Panel C and D) with fewer blood vessels developed. Furthermore, in the histopathological evaluation, the lungs of mutant mice had eosinophilic crystal deposition with evidence of pneumonia (MP:0001175). Furthermore, the spleen of mutant mice showed increased extra medullary hematopoiesis compared to the respective control mice.

*New findings from the GMC:* The *Bach2* KO mouse model from the GMC had bone and weight alterations indicative of reduced growth along with optic nerve disc abnormalities.

#### *Kl* (Klotho, Alpha)

Alpha *Klotho* KO mice were significantly lighter (*p* < 0.001, MP:0001262) and presented a shorter lifespan (MP:0002083) as they had to be sacrificed within 9 weeks of age due to welfare reasons. *Klotho* KO mice exhibited gait disturbances in the modified SHIRPA test (*p* = 0.015, MP:0001406) but were too weak to perform grip strength test. Also, KO mice showed decreased circulating glucose (*p* = 0.003, MP:0000189) along with increased calcium (*p* < 0.001, MP:0000194), inorganic phosphate levels (MP:0001566), and alkaline phosphatase activity (MP:0002968). Mutants showed increased circulating red blood cell number (MP:0002872) and hemoglobin levels (MP:0005564). Furthermore, a decrease in mean corpuscular volume (MP:0002591), mean corpuscular hemoglobin content (MP:0008956), and white blood cell number was detected. Multiple tissue mineralization (MPATH:555), characterized by deposits of calcium or calcium salts, was observed in trachea (Fig. 1 Panel E), heart, aorta, lung, kidney (Fig. 1 Panel F), and stomach (Fig. 1 Panel G) by positive Von Kossa staining at 9 weeks of age.

*New findings from the GMC:* The *Klotho* KO mouse model from the GMC had abnormal tissue mineralization in trachea, heart, aorta, lung, kidney, and stomach.

### Known rare disease genes: no pre-existing KO mouse models

#### *Kansl1* (KAT8 regulatory NSL complex subunit 1)

*Kansl1l* (KAT8 regulatory NSL complex subunit 1-like) KO mice were normally developed based on parameters of the phenotyping program. Eye alterations were observed in most of the *Kansl1l* mutants, mainly nuclear cataract with lenses displaying a unique ring-like structure and fiber cells leakage at the posterior lens cortex (10/18 mice, MP:0001304) and altered retina with reduced total thickness (6/18, MP:0011965). Mutant mice showed normal behavior except for a subtle decrease in prepulse inhibition (*p* = 0.01, MP:0009142). Blood analysis showed elevated sodium (*p* = 0.011, MP:0005633), iron (*p* = 0.026, MP:0005637) and calculated transferrin saturation (MP:0011897) and decreased calcium (*p* = 0.016, MP:0000195) plasma levels. The cardiovascular phenotyping of *Kansl1l* mutants observed increased left ventricular posterior wall end at systole (LVPWs, *p* = 0.021), decreased left ventricular internal diameter at end diastole (LVIDd, *p* = 0.031), and left ventricular (LV) mass (*p* = 0.035) as well as decreased stroke (*p* = 0.008) and cardiac output (*p* = 0.016) in mutant compared to control mice (Fig. 1 Panel H and I) allied with heart morphological alterations (fibrosis MPATH:181, vacuolation MPATH:825) in 3/4 mice in the histopathological study. In addition, *Kansl1l* mice showed morphological tissue alterations in kidney (cystic tubular dilation (MPATH:66), testis (MP:0001146), and epididymis (MP:0002631) of all mice investigated. The histopathological analysis clearly demonstrated strong links to the clinical chemistry and cardiovascular findings.

*New findings from the GMC:* The *Kansl1l* KO mouse model from the GMC was viable until 16 weeks of age with eye abnormalities such as nuclear cataract and abnormal retina thickness.

#### *Acsf3* (Acyl-CoA synthetase family member 3)

*Acsf3* KO mouse model of a RD of combined malonic and methylmalonic aciduria, presented significant mouse phenotypic traits related to an increased circulating iron (*p* < 0.001, MP:0008810) and decreased circulating unsaturated transferrin levels (*p* = 0.021, MP:0011897), suggestive of hemolytic anemia or liver disease. In addition, circulating levels of triglycerides (*p* = 0.01, MP:0005317) and urea were higher (*p* = 0.022, MP:0001552 and *p* = 0.01, MP:0005565), whereas calcium and phosphate levels were lower (*p* = 0.019, MP:0000195, *p* = 0.007, MP:0000198, respectively) in *Acsf3* mutant compared to WT mice. There was a reduction in the innate immune cell component in spleen cells (*p* = 0.039, MP:0002419) specifically in male *Acsf3* KO mice. In addition, more cluster differentiation (CD) CD62L + naive cell subset in the T cell population (*p* = 0.024, MP:0013436) was observed in the male mutants. Female *Acsf3* mutants had electrocardiography (ECG) alterations characterized by a prolongation in QRS (combination of the Q wave, R wave, and S wave) (*p* = 0.02, MP:0010392) and QT (Q wave to end of T wave) (*p* = 0.026, MP:0003233) interval lengths in structurally normal hearts, whereas males were similar. Increased circulating potassium (*p* = 0.006, MP:0005628), lipase (*p* = 0.042, MP:0011886) and urea (*p* = 0.011, MP:0005565) along with decreased bilirubin (*p* = 0.036, MP:0005635) levels were detected in female *Acsf3* mutants. In addition, platelet count was increased (*p* = 0.001, MP:0005505) in female *Acsf3* KO mice. Liver weights were increased in mutant compared to WT mice and in histology, focal hepatic alterations comprised of enlarged hepatocytes were found, showing tinctorially distinct, necrotic, binucleated and with intracytoplasmic inclusions, potentially indicating a process of cytotoxicity. In addition, mutant mice exhibited renal bilateral tubular hyperplasia and protein casts.

*New findings from the GMC:* The *Acsf3* KO mouse model from the GMC had abnormal innate immune cell components and higher T-sub cell, CD62L + naive cell, counts along with renal histopathological alterations.

#### *Pcdhgb2* (Protocadherin gamma subfamily B, 2)

The inactivation of *Pcdhgb2* gene was associated with increased volumetric bone mineral density (vBMD) in mice of both sexes (*p* = 0.008, MP:0013615) with no significant changes in areal BMD by DXA. There was an increase in the acoustic startle response (*p* = 0.03, MP:0001488) in mutant mice. They also exhibited a decrease in body fat mass (*p* = 0.045, MP:0014143). By TTE, decreased inner ventricular septal wall thickness in systole (IVSs, *p* = 0.038) and reduced left ventricular dimensions (left ventricular internal diameter at end systole (LVIDs), p = 0.046; LVIDd, *p* = 0.023; decreased LV mass (*p* = 0.048, MP:0010580)) were observed. Furthermore in ECG, a shortened P-wave (*p* = 0.039, MP:0011936) was observed in *Pcdhgb2* mutant compared to WT mice. Blood analysis showed that mutant mice exhibited lower albumin levels (*p* = 0.018, MP:0005419) and elevated amylase activity (*p* = 0.027, MP:0008806). In addition, decreased red blood cell count (*p* = 0.013, MP:0002875) and lower hematocrit (*p* < 0.001, MP:0001580) levels were observed in mutants compared to controls. At necropsy, at 16 weeks of age, heart and spleen weights were increased in mutant compared to control mice. Moreover, the splenic CD8 + CD62L + CD44 + cell population was increased (*p* = 0.009, MP:0010838). One mutant male had a bilateral hydronephrosis (MP:0000519) severe in the left kidney with total loss of renal tissue and presence of fibrosis in the histopathological examination. Two male mice had a preputial gland duct dilatation and 3/4 mutant mice an apparent mild increase in the left ventricular wall width.

*New findings from the GMC:* The *Pcdhgb2* KO mouse model from the GMC was viable until 16 weeks of age with alterations in LV dimensions.

#### *Rabgap1* (RAB GTPase activating protein 1)

The homozygous *Rabgap1* mice had lower body weight (*p* = 0.001, MP:0001262) compared to WT mice when sacrificed at 16 weeks of age. Male mutants showed** i**ncreased spontaneous locomotor (*p* < 0.001, MP:0031391) and exploratory (*p* < 0.001, MP:0001415) activities during the first five minutes of the open field test with increased resting time in the whole arena (*p* < 0.001, MP:0001402) along with decreased prepulsion inhibition (*p* < 0.01, MP:0009142) compared to control mice. There was a subtle reduction in forelimb grip strength (*p* = 0.01, MP:0010053) in mutant mice. The *Rabgap1* female KO mice had thinner retina (*p* = 0.015, MP:0011965) compared to control mice. The blood analysis results revealed, when compared to WT mice, improved glucose clearance (*p* < 0.001, MP:0005292), increased circulating urea (*p* = 0.043, MP:0005565), sodium (*p* = 0.009, MP:0005633), and alanine transaminase activity level (*p* = 0.012, MP:0002941) in mutant mice. In contrast, circulating cholesterol (*p* < 0.001, MP:0003983) was decreased in female mutants compared to control mice. In addition, female mutant mice also exhibited increased white (*p* = 0.036, MP:0000218) and red blood (0.006, MP:0003131) cell counts, hemoglobin content (*p* = 0.001, MP:0005564) and hematocrit (*p* = 0.001, MP:0000207) and decreased T effector cell in both spleen and peripheral blood (*p* = 0.04, MP:0002435). Histological analysis showed thinning of the corpus callosum (MP:0000780) in 3/4 mutant mice (Fig. 1 Panel J and K) and hydrocephaly (MP:0000916) in 1/4 mutant mice.

*New findings from the GMC:* The *Rabgap1* KO mouse model from the GMC offered a brain phenotype characterized by corpus callosum thinning and hydrocephaly.

#### *Cox7a2* (Cytochrome C oxidase subunit 7a2)

*Cox7a2* KO mice were apparently normal at birth but after weaning their health deteriorated (MP:0002083) and they were sacrificed at four weeks of age due to welfare reasons. At the time of sacrifice, mutant mice had lower body weight (*p* < 0.001, MP:0001262) than WT mice. Histological evaluation of several tissues revealed no apparent morphological tissue impairment except for an alteration in the morphology of the interscapular brown adipose and thymus tissues. Mutant mice exhibited larger lipid droplets in brown adipose tissue compared with control mice. On the contrary, *Cox7a2* heterozygous mice completed the whole phenotyping program (16 weeks) and exhibited no significant phenotypes when compared to WT.

*New findings from the GMC:* The *Cox7a2* KO mouse model from the GMC had a failure to thrive and adipose tissue abnormalities.

### Previously unreported RD gene candidates: novel mouse models

#### *Zdhhc5* (Zinc finger DHHC-type palmitoyltransferase 5)

**Fig. 2:**
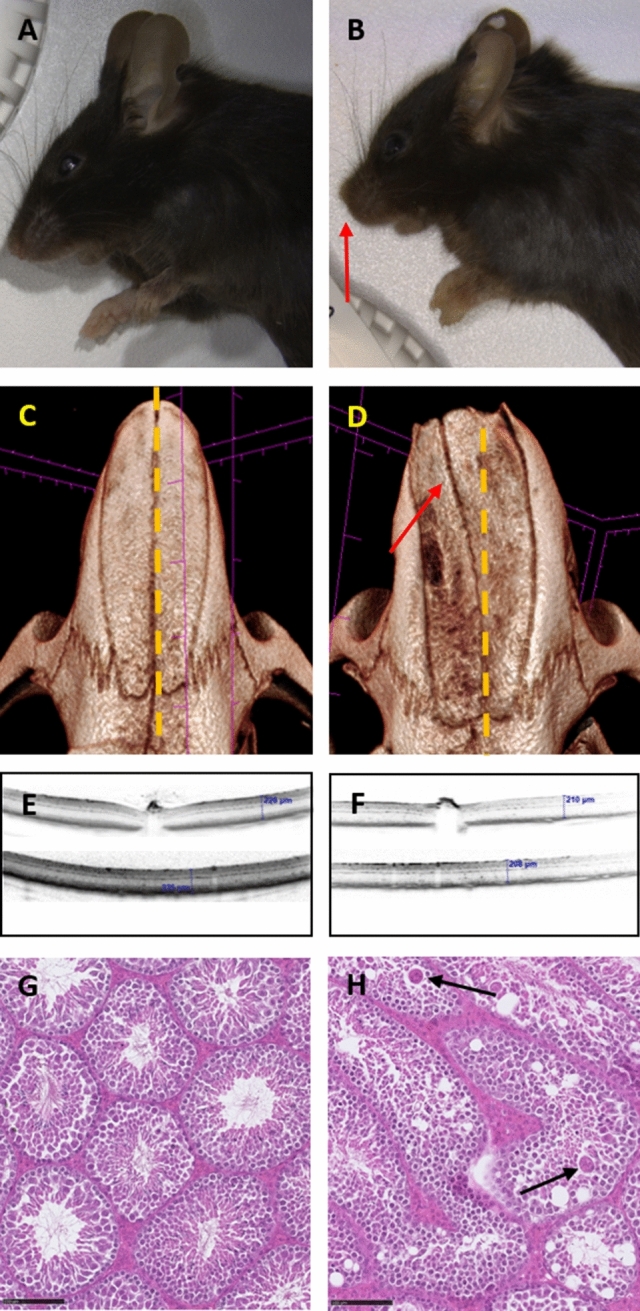
Representative high-throughput phenotyping findings from the GMC for *Zdhhc5* KO mouse model. Panel A-D: Macrophotographs (**A** and **B**) and micro computed tomography (**C** and **D**) imaging analyses show short snout and nasal bones (indicated with red arrow) with abnormal growth in *Zdhhc5* KO (**B** and **D**) in comparison with WT (**A** and **C**) mice. Panel E and F: Optical coherence tomography shows reduced retinal thickness in *Zdhhc5* KO (**F**) compared to WT mice (**E**). Panel G and H: Microphotographs of haematoxylin and eosin-stained testis show degenerative changes with vacuolated seminiferous tubules and multinucleated giant cells (black arrows) in *Zdhhc5* KO (**H**) compared to WT mice (**G**). All WT were of C57BL/6N background

Deletion of *Zdhhc5* (also known as *Dhhc5*) led to several different phenotypes. In X-ray analyses, a short snout (7 / 10, MP:0000445) with variable deviation in the skulls of KO mice of both sexes was observed (see macrographs Fig. 2 Panel A and B and micro computed tomography results in Fig. 2 Panel C and D). Acoustic startle response was decreased (*p* < 0.001, MP:0001489) particularly in the males. *Zdhhc5* male mutants had increased auditory brain stem response thresholds (*p* = 0.01, MP:0004738). In addition, *Zdhhc5* mice exhibited a reduced retinal thickness (*p* < 0.001, MP:0011965) (Fig. 2 Panel E and F) and most of the female mutants varying degrees of retinal layer alterations at the pigment epithelium possibly indicating formation of blood vessels in the normally avascular photoreceptor layer. KO mice had a reduced fat tissue content (*p* = 0.002, MP:0005454). This observation correlated with the decreased circulating cholesterol (*p* = 0.001, MP:0005179), triglyceride (*p* < 0.001, MP:0,002,644), and glycerol (MP:0,003,442) levels, predominantly in female mutant mice. In contrast, male mutants exhibited lowered albumin (*p* = 0.009, MP:0,005,419) plasma levels. Irrespective of sex, mutant mice showed slightly elevated potassium (*p* < 0.001, MP:0,005,627) and creatinine plasma levels (*p* = 0.019, MP:0005553) compared to WT mice. Elevated calcium (*p* = 0.049, MP:0000194) and phosphate (*p* = 0.028, MP:0001566) and decreased glucose (*p* = 0.001, MP:0001566) plasma levels were also observed in mutant mice. In addition, increased mean platelet volume (*p* < 0.001, MP:0002599) and platelet distribution width due to high proportion of large platelets (*p* < 0.001, MP:0002599) can indicate coagulopathy with increased risk of cardiovascular disease. Subtle erythrocytic macrocytosis was shown by elevated mean corpuscular volume (*p* < 0.001, MP:0002590) and mean corpuscular hemoglobin (*p* = 0.028, MP:0005561) in *Zdhhc5* mutant mice. In ECG, *Zdhhc5* mutant mice had increased heart rate (*p* = 0.031, MP:0002626), shortened PR length (*p* = 0.001, MP:0010511), and prolonged QRS (*p* = 0.025, MP:0010392) and QT intervals (*p* = 0.027, MP:0003233). Mutants had increased heart weight (*p* = 0.011, MP:0002833) normalized to body weight compared to controls. Two male mutant mice had unilateral hydronephrosis (MP:0000519) based on histological analysis. Furthermore, both males had degenerative changes in the testes with vacuolated seminiferous tubules and multinucleated giant cells (Fig. 2 Panel G and H). One male mutant showed reduced sperm content in the cauda of the epididymis due to absent sperm maturation in the epididymis (MPATH 797).

#### *Wsb2* (WD repeat and SOCS box containing 2)

The *Wsb2* KO mice presented with abnormal upper teeth (MP:0002100) with smaller maxillary incisors in comparison with WT. There was a decrease in bone mineral content in male KO mice (*p* = 0.007, MP:0010124). Concurrently, body weight (*p* = 0.001, MP:0001262) and fat mass (*p* = 0.012, MP:0014143) were decreased in male mutants at reduced food intake during calorimetry (*p* = 0.005, MP:0011940). They showed decreased rearing activity (*p* = 0.007, MP:0002757) and decreased acoustic reactivity (*p* < 0.001, MP:0001488). Furthermore, *Wsb2* mutants showed increased locomotor activity (*p* = 0.03, MP:0031391) and more tail elevation. In comparison with WT mice, KO *Wsb2* mice exhibited reduced retinal thickness (*p* < 0.001, MP:0011965) and reduced number of fundic main blood vessels (*p* < 0.001, MP:0002792) (Fig. 1, Panel N and O). They had increased plasma potassium (*p* = 0.029, MP:0005627) chloride (*p* = 0.05, MP:0003019), iron (*p* = 0.008, MP:0008810), alkaline phosphatase (*p* < 0.001, MP:0002775), alanine transaminase (*p* = 0.021, MP:0002941), and aspartate transaminase (*p* = 0.007, MP:0005343) levels when compared to WT. Additionally, there were lower glucose levels (*p* = 0.04, MP:0005560) in both sexes, significantly reduced phosphate (*p* = 0.009, MP:0000198) and increased insulin (*p* = 0.003, MP:0002079) levels in male mutants in comparison to control mice. TTE showed reduced inner ventricular septal wall thickness (*p* = 0.029, MP:0006113) in *Wsb2* male mice and reduced heart rate (*p* < 0.001, MP:0005333) and less cardiac output (*p* = 0.05, MP:0003393) at increased respiratory rate (*p* = 0.01, MP:0005573) in mutant mice of both sexes when compared with WT mice. In the histological examination, severe testicular tubular atrophy and Leydig cell hyperplasia, were evident in male *Wsb2* KO mice. In addition, persistence of adrenal cortical X-zone, reduced number of convoluted tubules in the submandibular salivary gland and partial absence of parietal epithelial cells lining Bowman’s capsule in the kidneys was detected. Of note, expression of lacZ was observed in gray matter neurons of the spinal cord, brain, cornea, lens, sclera, anterior part of the pituitary, urothelium, sweat glands, testis, cartilages of ribs and sternum, terminal bronchioles of the lung and glandular epithelium of the stomach.

## Discussion

Mouse production and comprehensive phenotypic analysis through standardized protocols successfully allows the GMC, as part of the IMPC, to build a variety of phenotypic annotations in KO mouse lines. These are valuable traits in the mouse model to mimic RD phenotypes in humans.

From a large repository of data in the GMC, we selected representative examples of (1) genes when deleted (KO mouse) mimic some characteristics associated with known RDs, (2) Known RD genes with no pre-existing KO mouse models, and (3) genes with previously unknown functions and disease associations that may represent future RD models. These groups provide mouse models for known RDs or highlight new, previously unknown phenotypic annotations describing a potentially novel function for a gene of interest, and thus the possibility for further experimentation in multifaceted RDs.

### Known rare disease genes: KO mouse models

#### *Nacc1* (Nucleus accumbens associated 1)

NACC1 is part of the BTB/POZ domain-containing family of genes and the encoded protein is a transcriptional repressor that regulates, among other processes, proliferation and apoptosis (Ishibashi et al. 2009; Korutla et al. 2009). It is expressed in the adult central nervous system in neurons with an established influence on synaptic plasticity (Mackler et al. 2000; Shen et al. 2007). The mechanistic role played by NACC1 in brain development is not yet characterized in detail. Patients suffering from Neurodevelopmental disorder with Epilepsy, Cataracts, Feeding difficulties, and delayed brain Myelination (NECFM, ORPHA: 500545) have a de novo heterozygous mutation (c.892C > T) in the *NACC1* gene. The study of (Schoch et al. 2017) reported seven children with microcephaly, profound developmental delays and/or intellectual disability, hypotonia, cataracts, severe epilepsy including infantile spasms, irritability, failure to thrive, and stereotypic hand movements. The case reports indicated that two of the seven probands showed also bilateral sensorineural hearing loss. Based on the analyses performed to date, *Nacc1* deficiency in the mouse led to phenotypes in agreement with those found in patients. These included abnormal sensory processing (under responsive to mild environmental stressors, sensorineural hearing loss) in line with the sensory processing dysfunction described along with the core symptoms of neurodevelopmental disorders such as autism (Marco et al. 2011). Furthermore, consistent with the patient hypotonia and failure to thrive, the *Nacc1* KO mice showed motor impairment and perturbed growth. A more in-depth characterization of the model will reveal whether these alterations align with the impaired cognition, myelination defects, and excitation/inhibition imbalance evidenced in the patients. A novel finding in our viable *Nacc1* KO model was the renal abnormality, associated with alterations in the blood chemistry profile. This phenotype was not described previously for the gene and (Schoch et al. 2017) did not identify, or at least assess, kidney dysfunction in the patients with NACC1 mutations. Nevertheless, NACC1 is expressed in the kidney and this work now highlights the pleiotropic functions of this gene in brain and kidney. The dysmorphological features of mutant mice in this and other studies (Yap et al. 2013) (B6) together indicate an important role of *Nacc1* gene on vertebral number variation and bone development and homeostasis in mice. Moreover, the authors reported that fewer *Nacc1*^−/−^ newborn mice survived until weaning, suggesting a role of the gene during prenatal and early postnatal development.

Summarizing, we have been able to show, in addition to neurodevelopmental disorder-related defects, a clear renal injury of high penetrance in our viable *Nacc1* KO mouse model. Thus, in terms of added translational value, this preclinical functional assessment has disclosed the potential for renal comorbidity to exclude in *NACC1* mutation-carrying patients. The parallel generation of a precision model using the patient-specific variant (e.g., c.892C > T identified by Schoch et al., 2017) will aid in elucidating the protein region involved and hence the likelihood of patient vulnerability to kidney dysfunction. It is currently not clear if a direct relationship exists between the renal and the brain function-derived *Nacc1* KO-induced abnormality, however this established functional model can potentially yield insights in this regard.

#### *Bach2* (BTB and CNC homology 1, basic leucine zipper transcription factor 2)

Chronic Myeloid Leukemia (CML, ORPHA:521) is associated with *BACH2* deficiency. Recently, (Zhang et al. 2022) reported that the expression of IRF4 and BACH2 is notably down regulated in CML patient T cells. Lymphocyte maturation defects and upper respiratory chest infections have been reported in patients with *BACH2 *heterozygous mutations (Afzali et al. 2017). This study reported three patients from two families with inflammation of the large bowel, lung inflammatory infiltrates and recurrent upper respiratory tract infections together with splenomegaly. Taking into account the high throughput analyses performed in the GMC, *Bach2* deficiency in the mouse led to phenotypes that associate to those reported in these patients. For instance, the CRS-deficiencies detected in *Bach2* KO mouse splenocytes. It has been shown that CSR-deficiencies are highly associated with a DNA repair factor deficiency, and that leads to the occurrence of lymphomas and leukemia (Durandy et al. 2007). In addition to the immunophenotype, phenotypes such as histopathological alterations in the lung, spleen, and eye were observed in mutant mice. These included eosinophilic crystal deposition with evidence of pneumonia, increased extra medullary hematopoiesis and optic nerve dysmorphology, respectively. Recent human genetic studies indicated a genetic polymorphism within the *BACH2* gene locus and its association with autoimmune conditions such as autoimmune thyroid disease, Addison’s disease, systemic lupus erythematosus among others and diabetes type 1 (Fichna et al. 2021). In fact, BACH2 variants were associated with diabetes type 1 in a study of genome-wide association (GWA) genotyping (Grant et al. 2020) and treatment with a BACH inhibitor lowered glycemia in a model of single cells of pancreatic islets from diabetes type 2 individuals (Son et al. 2021). KO of the *Bach2* gene in our center revealed decreased plasma glucose levels, which is in agreement with the results of the islets study. In addition, *BACH2* is referred as a candidate gene in a profiling study of patients with myocardial infarction (Qiu and Liu 2019). It is evident that *Bach2* KO mouse phenotypes share many clinical similarities with patients suffering from CML. As a RD myeloproliferative disorder, it is a remarkable correlation between the immunological, respiratory and splenic pathological phenotypes observed in the GMC KO model. Interestingly, *Bach2* KO mice showed increased liver enzyme activities, namely aspartate transaminase, considered to be a myocardial infarction biomarker in humans. Previous homozygous mouse models have made substantial contributions to understanding the role of *Bach2* gene in controlling autoimmunity (Zhang et al. 2019), pulmonary alveolar proteinosis (Nakamura et al. 2013), and tumor immunosuppression (Roychoudhuri et al. 2016). By using the current GMC study layout, our *Bach2* KO model adds further behavioral characterization and eye phenotypes.

In summary, we establish *Bach2* KO mice as a new and versatile model to explore the underlying physiological mechanisms of abnormal hematopoietic maturation and its overall therapeutic potential.

#### *Kl* (Klotho, Alpha)

Loss of KLOTHO ALPHA expression is associated with Familial hyperphosphatemic tumoral calcinosis/hyperphosphatemic hyperostosis syndrome (ORPHA:306,661). An affected 13-year-old girl with a homozygous loss of function *KLOTHO* mutation presented severe tumoral calcinosis, carotid artery calcifications, hypercalcemia, and high renal tubular reabsorption of phosphate (Ichikawa et al. 2007) as observed in the GMC *Klotho* KO mice. The mice were too weak to remain in the pipeline testing and perform the grip strength test, which has been previously reported in transgenic *Klotho* mice on different background strains (mixture of C57BL/6 J and C3H/J and also BALB/c) (Kuro-o et al. 1997). The authors and others (background strain of C57BL/6 and C3H) have clearly demonstrated elevated calcium and phosphorus levels in *Klotho* mutant mice (Tsujikawa et al. 2003). In addition, the GMC mutant line displayed a hematological phenotype, which confirms the mouse study of Vadakke Madathil et al. 2014. *Klotho* mutant mice present a bone phenotype (Kawaguchi et al. 2000) as expected from the increased systemic calcium and phosphate as previously reported (Kuro-o et al. 1997). The observed histopathological tissue mineralization in several tissues is in agreement with the disturbance in mineral metabolism, particularly with progressive renal disease due to altered calcium-phosphorus metabolism.

In summary, our data serve as a proof-of-concept model for a single gene-causing familial tumoral calcinosis due to a relation to hypercalcemia, hyperphosphatemia, and distinct histological-evidence of diverse tissue calcifications.

### Known rare disease genes: no pre-existing KO mouse models

#### *Kansl1* (KAT8 regulatory NSL complex subunit 1)

Koolen-de Vries syndrome (ORPHA:96,169) is a RD characterized by visual impairments, cardiovascular defects, renal and urogenital anomalies, which have been individually highlighted in a cohort of 45 patients with either a 17q21.31 microdeletion encompassing the KAT8 regulatory NSL (non-specific lethal) complex subunit 1 gene or a truncating variant in the *KANSL1* gene scattered throughout the gene (Koolen et al. 2016). “*Males with Koolen-de Vries syndrome often have undescended testes (cryptorchidism). Defects in the walls between the chambers of the heart (septal defects) or other cardiac abnormalities, kidney problems, and skeletal anomalies such as foot deformities occur in some affected individuals*” (MedlinePlus). The study of (Tan et al. 2009) examined previously unreported eleven patients with the 17q21.31 microdeletion showing hearing, cardiac and renal impairments. Interestingly, (Lalani 2020) reported that the cardiac phenotype in humans is probably associated to an imbalance of other genes within the deleted interval as congenital heart disease was not found in individuals with single nucleotide variations in *KANSL1*. Homozygous *Kansl1 KO* mice are not viable (embryonic lethal (Li et al. 2022)) and therefore homozygous *Kansl1l* mutant mice were generated as a viable *Kansl1l* KO model in the GMC. It is striking that the phenotypes observed in *Kansl1l*-disrupted mice, including decreased retinal thickness and hearing loss, were present in 14% and 43% of human patients despite the age among the patients being variable (Farne et al. 2022). The same study reported that other phenotypes such as cardio and renal defects were observed in 24% and 9% of human cases, respectively. However, dysmorphic features and anemia found in humans were not observed in *Kansl1l* KO mice. Overall, the *Kansl1l* data indicated many phenotypic similarities in organs, such as heart, kidney, testes, and eye, between the KO mouse and patients suffering from the rare monogenic Koolen-de Vries syndrome (Moreno-Igoa et al. 2015) (Koolen et al. 2016) (Amenta et al. 2022). Moreover, what is noteworthy from the literature is that the few mouse models so far published relate to a *Kansl1* heterozygous variant (Arbogast et al. 2017).

Summarizing, the viable *Kansl1l* KO mouse clearly demonstrates strong associations with clinical chemistry, cardiovascular and renal phenotypic descriptors based on histopathology and offers the possibility to elucidate in-depth heart and renal abnormalities in these patients.

#### *Acsf3* (Acyl-CoA synthetase family member 3)

Several studies have associated *ACSF3* deletion to Combined Malonic And Methylmalonic Aciduria (CMAMMA, ORPHA: 289504) (Sloan et al. 2011; Wang et al. 2021). This RD presents heterogeneous clinical manifestations in patients that vary from anemia, hepatomegaly, myocardial and liver damage (Wang et al. 2021). Moreover, young patients with mutations affecting the *ACSF3* gene, diagnosed with combined malonic and methylmalonic acidemia via newborn screening, may present no clinical symptoms or signs indicative of a metabolic disorder at a young age. This suggests that *ACSF3* deficiency may be a benign condition unless triggered by other factors (Levtova et al. 2019) (Wang et al. 2021). The phenotypic characterization of *Acsf3* KO mice exposed liver abnormalities, which were more pronounced in female than in male mutant mice. The histopathological evaluation revealed increased liver weight with distinct morphologically altered hepatocytes, suggestive of a mild process of cytotoxicity and renal tubular hyperplasia with protein casts when compared with controls. The altered circulating levels of iron, unsaturated iron binding capacity, urea, triglycerides, and bilirubin further indicate metabolic alterations. In addition, there were observations of impaired heart function and hyperkalemia, which in combination may indicate a possible degradation of heart function and conduction system. The study of (Tucci 2020) highlighted the urgency of an *ACSF3* deficient mouse model for studying the first step of the mitochondrial fatty acid biosynthesis (mtFASII) pathway (activation of malonic acid to malonyl-CoA) on cellular energy maintenance in neural cells. In addition, the *Acsf3* KO model indicated novel findings, including reduction in the innate immune cell component and renal alterations associated with changes in the blood chemistry profile that can aid in determining if the patients are at risk of other identical lesions as seen in the gene KO mouse model and should be further investigated.

Here, we highlight the importance of KO mouse models in simulating known human phenotypes and in revealing new tangential renal and immunology phenotypes worth exploring.

#### *Pcdhgb2* (Protocadherin gamma subfamily B, 2)

*PCDHGB2* gene is associated with the very rare Prune Belly Syndrome (ORPHA 2970), where patients exhibit deformations in the musculoskeletal, renal and urinary systems. The gene belongs to a protocadherin gamma gene cluster (*PCDH-GAMMA*), which encodes 22 calcium-mediated cell adhesion molecules that mediate neural cell-to-cell connectivity. In another gamma-protocadherin, *PCDHGA3*, the gene expression levels relate to ventricular dysfunction in patients with ischemic cardiomyopathy (Ortega et al. 2016), indicating the role of this protein cluster for heart functionality. In our study, *Pcdhgb2* KO mice showed mild renal and cardiovascular phenotypes substantiated by the lower albumin and elevated amylase blood levels, anemia, increased T cells, severe hydronephrosis in one mutant mouse and the reduction of LV cavity due to a thickening (e.g., hypertrophy) of the heart wall. Deletion of the complete *Pcdh*-gamma cluster in mice yielded neuronal apoptosis in which the mice did not survive after birth (Wang et al. 2002), suggesting the role of the gamma gene cluster in coordinated movement and neonatal lethality.

To the best of our knowledge, the *Pcdhgb2* KO model is the first in vivo model and thus important to study the interconnected phenotypic presentation of renal and cardiac pathophysiology, paralleled by clinical sequencing efforts in patients with clinical manifestations.

#### *Rabgap1* (RAB GTPase activating protein 1)

Warburg Micro Syndrome (WARBM, ORPHA: 2510), also known as Micro Syndrome, is a RD caused by mutations in *RAB3GAP1* (Morris-Rosendahl et al. 2010) (Tasdemir et al. 2015) and *TBC1D20* (Liegel et al. 2013) genes. Among other characteristics, individuals homozygous for *RAB3GAP1* present failure to thrive, intellectual disability, corpus callosum hypoplasia, diffuse cortical or subcortical atrophy, congenital cataracts, microcornea and microphthalmia (Morris-Rosendahl et al. 2010). *TBC1D20* mutation (TBC1D20 protein as a GTPase-activating protein (GAP) for RAB1 and RAB2) causes cataracts and male infertility in patients with WARBM (Liegel et al. 2013). In this study, *Rabgap1* KO mice presented features that resemble growth deficiency and brain, eye and neurological phenotypes in agreement with the WARBM clinical manifestations (Oh et al. 2022). In addition, histological analysis showed thinning of the corpus callosum and hydrocephaly in mutant mice. The study by (Liegel et al. 2013) reported that *blind sterile* (*bs*) mice (loss of function *Tbc1d20* mutation, AKR/J background) also exhibited eye and testicular phenotypes but no morphological alterations in the brain. In addition, silencing *Rabgap1* in mouse fibroblasts led to the intracellular accumulation of active beta1 integrins that not only altered focal adhesion formation but also decreased cell migration and cancer cell invasion in vitro (Samarelli et al. 2020). The authors further reported that increased *Rabgap1* mRNA levels were associated with a poor prognosis in human breast cancer patients.

Overall, the *Rabgap1* KO mouse model presents interesting histopathological thinning of the corpus callosum, recapitulates the major features of WARBM patients and adds translational value contributing to treatment efforts.

#### *Cox7a2* (Cytochrome C oxidase subunit 7a2)

In humans, the gene *COX7A2* is associated with Fatal Infantile Cardioencephalomyopathy due to Cytochrome C Oxidase Deficiency (ORPHA1561), a very rare mitochondrial disease, clinically recognized by cardioencephalomyopathy resulting in death in infancy. Literature in human related to *Cox7a2* gene is scarce. The phenotypic characterization of *Cox7a2* KO mice was not possible as the mice health deteriorated soon after weaning and only terminal tests were performed. A histopathological evaluation revealed mild alterations in brown adipose tissue and thymus morphology. The abnormal body weight and brown fat morphology at sacrifice, probably through damaged mitochondrial function, may have caused a gradual decline in the thermogenic capacity of the *Cox7a2*-disrupted mice in the cold (after weaning). In fact, in another *Cox7a* gene model, adult mice KO for cytochrome c oxidase subunit 7a-related polypeptide (*Coxrp*), also known as *Cox7a2l*, exhibited muscle weakness and impaired thermogenesis in the cold, despite normal body weight when compared to WT mice (Ikeda et al. 2013). In addition, the authors reported that mutant mice exhibited a decreased metabolic rate in the steady state and during exercise and a metabolic shift to glucose consumption during treadmill exercise. Another study (Shiba et al. 2017) showed that *Cox7a2l* KO mice had decreased blood glucose levels after insulin or pyruvate challenge, further pointing to the role of *Cox7a2l* in glucose homeostasis. It has been reported (Maurer et al. 2015) that Cox7a1 and Cox7a2 isoforms are of identical importance for cytochrome c oxidase activity in brown adipose tissue and heart. In another mouse study, *Cox7a1* KO mice were viable and presented a dilated cardiomyopathy at 6 weeks of age with the *Cox7a1* KO mice incorporating more of the “liver-type” isoform of *Cox7a2* into the cardiac Cox holoenzyme, translating in higher tissue ATP levels (Huttemann et al. 2012). Therefore, it is plausible that the GMC *Cox7a2* homozygous mice suffered from perturbed glucose and energy homeostasis via impaired oxidative phosphorylation (complex IV of the electron transport chain). In early postnatal relevant tissues, such as brown adipose tissue and liver, *Cox7a2* gene might function therefore as the limiting factor for the mice survival after weaning. There was clear histological-evidence of altered brown adipose tissue morphology in KO *Cox7a2* mice. Furthermore, their lower body weight and premature death highlight the importance of gene editing tool to study rare mitochondrial diseases that probably cause the death of neonatal patients. The difference in the described phenotypes between the *Cox7a2*-disrupted mice and infants harboring *COX7A2* mutations could have resulted from differences in protein expression required for normal activity of mitochondrial respiration in high energy-demanding tissues such as the heart and brain. In a recent study by (Benegiamo et al. 2022), the authors showed that Cox7a2l was induced in skeletal muscle upon exercise in mice and that humans with higher COX7A2L expression had better metabolic and cardiorespiratory phenotypes. The study of (Wojcik et al. 2019) reported that 47% of the infant deaths exhibited confirmed monogenic conditions with a great proportion due to inborn errors of metabolism.

### Previously unreported RD gene candidates: novel mouse models

We have experimentally identified gene-related phenotypes that are not yet associated with known human RDs. In our KO mouse models, however, we show genotype–phenotype links suggestive of gene causality. Aligning these observations from mouse to man is currently impossible due to missing human RD data.

#### *Zdhhc5* (Zinc finger DHHC-type palmitoyltransferase 5)

Recently, there has been an increased interest on the regulatory role of palmitoyltransferase enzymes in many cell types including cardiomyocytes (Woodley and Collins 2021) and neurons (Shimell et al. 2021). According to the latter study, *DHHC5* protein is highly expressed in brain and moderately in heart and liver. There is no reported association of *ZDHHC5* (or *DHHC5*) gene with a rare or common disease at present, but it has recently been associated with sociability in the general population in a GWAS study involving 342,461 adults in the UK biobank, indicating that variants of this gene could potentially contribute to several psychiatric disorders (Bralten et al. 2021). The number of functional alterations observed in the present KO mouse model, however, shows a gene with organ-specific roles. Our battery of phenotypic tests showed links to neural sensory processing abilities and/or hearing defects, retinal morphology, cardiovascular and renal diseases. In the present *Zdhhc5* KO mouse model, the perturbed cardiac function and the increased potassium and calcium circulating levels highlight the mouse as a suitable model to explore cardiac muscle phenotypes. In fact, (Lu et al. 2019) reported a role of *DCCH5* in modulating Na/K pump densities and cardiac contractibility in murine cardiac myocytes. In addition, it is known that *ZDHHC5* is a palmitoyltransferase that modulates the activity of many proteins, some of them known cardiovascular disease-causing genes such as desmosomal cadherin desmoglein-2 (*DSG2*) and myelin regulatory factor (*MYRF*) (Woodley and Collins 2021). Furthermore, while our GMC KO mice displayed merely impaired auditory processing, another *Zdhhc5*-GT (gene trapping on C57BL/6 background) mouse model has elucidated alterations in hippocampal learning (Li et al. 2010) and in excitatory synapse formation (Shimell et al. 2021) at 10–12 weeks of age. The observation that *Zdhhc5* KO mice exhibited craniofacial and eye phenotypes are novel findings. Here, we offer a new and exciting KO mouse model potentially useful for study of palmitoyltransferase enzymes deficiency in the first years of life, in which the patients exhibit weakened muscle and heart, brain, kidneys, and facial abnormalities. In addition, histological analysis revealed hydronephrosis and male gonadal morphological alterations, which, associated with high circulating values of potassium and creatinine in the clinical chemistry phenotypic analysis, may elucidate genotype-specific kidney disease. The identification of skull, cardio, and renal-related phenotypes in the *Zdhhc5* mouse KO model should prompt further investigations in mice and human, given the likelihood of potential association with other proteins some of which are known to be disease causing. Finally, in an aged cohort (data not shown), *Zdhhc5* KO mice presented prevalent short snout and ambiguous genitalia confirming our early onset pipeline findings. With 74 weeks of age, *Zdhhc5* KO mice exhibited eye, cardio, and clinical chemistry phenotypes.

The range of phenotypes in the *Zdhhc5* KO model accentuates the importance of this gene. Key phenotypes and their biological meaning in disease should be further elucidated to evaluate novel sex-related differences in therapeutic approaches of not yet identified rare human diseases.

#### *Wsb2* (WD repeat and SOCS box containing 2)

We generated the first *Wsb2* KO mouse model. These mice showed abnormalities in tooth morphology, locomotor activity, retina, heart, osmotic and electrolyte balance, all together hinting toward impaired renal and/or cardio function (secondary effect), and male infertility. Interestingly, the histopathological tissue examination found the presence of adrenal cortical X-zone in male *Wsb2* mutant mice. Of note, the normal regression of the X-zone in male mice normally occurs at puberty in C57BL/6 strain mice and perhaps the testicular atrophy, and the lack of gonadal hormones, prolonged its persistence in this study. These morphological abnormalities along with the Bowman’s capsule in glomeruli and submandibular salivary gland ductal changes are highly suggestive of a defective androgen-receptor response or androgen synthesis or action or both.

There is no rare or common disease associated with the *Wsb2* gene. This gap led us to hypothesize that there are potential corresponding clinical changes that may occur in a human disease not yet identified. The *Wsb2* gene may be a future RD candidate and the mouse KO model may recapitulate characteristics of a yet unidentified RD.

In this report, we demonstrate that knocking-out a single gene in mice is an important tool to gain in vivo innovative phenotypic information. The KO mouse models generated at the GMC/IMPC, however, are by nature a very radical model (e.g., full body knockout) and on one single background strain (C57BL/6N) at similar age. It is known that the severity of certain phenotypes may depend on the interaction of the mutation of interest with genetic factors of the mouse background as reported in diabetic mouse models (Coleman 1982) and others (Schughart et al. 2013). Humans have a natural variation in their genetic makeup and different age of disease onset. These differences between species may lead to some discrepancy. Nevertheless, these mice offer a first hint toward gene-disease causality and support in-depth “*dissection of the interplay of pathological and physiological mechanisms*” (Brown 2021).

In summary, high-throughput phenotyping of KO mouse models provides a width of data across all organ systems important for disease understanding and modeling. Using state-of-the-art technology at the GMC (https://www.mouseclinic.de/screens/overview/index.html), we have successfully produced comprehensible and accessible phenotypic data to recapitulate monogenic RD phenotypes. In this report, we highlighted known rare disease genes (e.g., *Nacc1, Bach2, Klotho alpha*) and aligned the results with previously published human and mouse studies. Where appropriate, we have emphasized novel findings that may add new biological function for the gene of interest, optimizing clinical diagnosis. The alignment of patient clinical characteristics to the comprehensive catalogue of parameters in the mouse is often missing in RD studies. We consider the histopathological findings of a wide variety of tissues to be a particular strength in the mouse models. It can be correlated with phenotypic characterization in vivo, confirm phenotypes and contribute to a deeper identification of the phenomenon. This achievement may be difficult in a clinical setting; therefore, pathological evaluation of mutant mice is instrumental for deeper understanding of human RD and offers particular value for pre-clinical testing. Simultaneously, we presented recognized RD genes with no pre-existing KO mouse models (*Kansl1l, Acsf3, Pcdhgb2, Rabgap1, Cox7a2*) and highlighted their importance in simulating human phenotypes. Concurrently, this report reveals new tangential phenotypes worth exploring among the research community. In our last category of genes, the GMC KO *Zdhhc5* and *Wsb2* mouse models represent an effort necessary to identify phenotypes in genes that are not presently associated with known human RDs.

In many cases, RDs have some unique challenges, particularly the genetic variants, difficulty of diagnosis, phenotypic heterogeneity and limited patient number in a complex disease. There are many undiagnosed RDs where a genetic origin is suspected. There is still a large lack of understanding for part of the genome and its association with phenotypic patterns. Therefore, it is this gap that leads to a lack of understanding in RDs. Thus, the KO mouse studies represent an effort necessary to support RDs research by performing standardized phenotypic characterization of mice, which are designed to have a deleted gene and its associated phenotypes, overcoming the bottlenecks in the human population. The KO mouse is therefore a resourceful translational model to explore the pathogenesis of RDs, gene causality and assist the evaluation of new therapeutic treatments (gene therapy) and interventions.

In conclusion, here, we offer insights into the GMC research efforts into monogenic causes of RDs in humans. The collaborative alliance with geneticists, clinicians and patient groups will have the potential to accelerate the development of treatments, that will ultimately help more patients in the best possible way. Reaching out to a collaborative work approach with genetic screening clinicians, for example via Genematcher (https://genematcher.org/statistics/) and Modelmatcher (https://www.modelmatcher.net/), the KO mouse model can support the foundation of new RD research programs and improve the “gene-to-patient” approach (Seaby et al. 2021), consequently promoting the discovery of new RD gene targets.

## Data Availability

All data are publically and freely available on the GMC (www.mouseclinic.de/results/phenomap-and-results/index.html) and on the IMPC portal (https://www.mousephenotype.org/data/search).
